# Feasibility of Cardiac Rehabilitation Models in Kenya

**DOI:** 10.5334/aogh.3392

**Published:** 2022-01-18

**Authors:** G. Titus K. Ngeno, Felix Barasa, Jemimah Kamano, Edith Kwobah, Charity Wambui, Cynthia Binanay, Joseph R. Egger, Peter S. Kussin, Nathan M. Thielman, Gerald S. Bloomfield

**Affiliations:** 1 Duke University, Durham, NC, US; 2 Moi Teaching and Referral Hospital, Nandi Road, Eldoret, KE

## Abstract

**Background:**

Heart failure (HF), is a leading cause of cardiovascular morbidity and mortality in Sub-Saharan Africa. Cardiac rehabilitation (CR) is known to improve functional capacity and reduce morbidity associated with HF. Although CR is a low-cost intervention, global access and adherence rates to CR remain poor. In regions such as Western Kenya, CR programs do not exist. We sought to establish the feasibility CR for HF in this region by testing adherence to institution and home-based models of CR.

**Methods:**

One hundred participants with New York Heart Association (NYHA) class II and III HF symptoms were prospectively enrolled from a tertiary health facility in Western Kenya. Participants were non-randomly assigned to participate in one of two CR models based on their preference. Institution based cardiac rehabilitation (IBCR) comprised 36 facility-based exercise sessions over a period of 12 weeks. Home based cardiac rehabilitation (HBCR) comprised weekly pedometer guided exercise targets over a period of 12 weeks. An observational arm (OA) receiving usual care was also enrolled. The primary endpoint of CR feasibility was assessed based on study participants to adherence to at least 25% of exercise sessions. Secondary outcomes of change in NYHA symptom class, and six-minute walk time distance (6MWTD) were also evaluated. Data were summarized and analyzed as means (SD) and frequencies. Paired t-tests, Chi Square, Fisher’s, and ANOVA tests were used for comparisons.

**Findings:**

Mean protocol adherence was greater than 25% in both CR models; 46% ± 18 and 29% ± 11 (P < 0.05) among IBCR and HBCR participants respectively. Improvements by at least one NYHA class were observed among 71%, 41%, and 54%, of IBCR, HBCR and OA participants respectively. 6MWTD increased significantly by a mean of 31 ± 65 m, 40 ± 55 m and 38 ± 71 m in the IBCR, HBCR and OA respectively (P < 0.05).

**Conclusions:**

IBCR and HBCR, are feasible rehabilitation models for HF in Western Kenya. Whereas improvement in functional capacity was observed, effectiveness of CR in this population remains unknown. Future randomized studies evaluating effect size, long term efficacy, and safety of cardiac rehabilitation in low resource settings such as Kenya are recommended.

## Background

Cardiovascular disease is a major driver of global morbidity and mortality, accounting for approximately 50% of non-communicable disease deaths worldwide [1]. Low and middle income countries account for over 80% of global cardiovascular disease mortality [2], with heart failure (HF) manifesting as a terminal complication. In Sub-Saharan Africa, HF afflicts mostly young and economically active adults and leads to severe impairment in quality of life, and loss of productivity amongst patients, their families and society in general [3,4].

Cardiac rehabilitation (CR) is a multidisciplinary approach providing physical, psychological and social support to patients recovering from cardiac illnesses such as HF. CR typically involves structured exercises based on an exercise prescription, lifestyle modification, counseling and health education [5]. Amongst patients with HF, CR has been shown to have multiple benefits including reduced hospital readmissions, improved exercise capacity and improvement in overall quality of life [5,6]. There are two common models for delivering CR: institution-based cardiac rehabilitation (IBCR), and home-based cardiac rehabilitation (HBCR). IBCR and HBCR models are similar in efficacy, and to have comparable low risk profiles [7,8].

Despite the known benefits of cardiac rehabilitation, global uptake has been slow. It is generally under-prescribed and has low adherence rates [6]. Commonly cited barriers to utilization, and drivers of early participant dropout, are poor referral systems, and inaccessibility of rehabilitation centers [6,9]. With the exception of high income urban centers, there has been little development of CR in in sub-Saharan Africa [10]. In regions such as Western Kenya, where the burden of HF disease is high, CR programs are non-existent [11].

## Objective

This study sought primarily to evaluate the feasibility of IBCR and HBCR models among patients with HF in Western Kenya, by measuring the ability of study participants to adhere to at least 25% of CR protocol sessions in either model. The study secondarily sought to assess functional capacity changes as measured by change in New York Heart Association (NYHA) class [12] and six minute walk time distance (6MWTD) [13] among patients with HF participating in either of the two models of CR.

## Methodology

This was a prospective, non-randomized, parallel assessment interventional study of patients engaging in IBCR or HBCR. The study was conducted at the Moi Teaching and Referral Hospital (MTRH), Uasin Gishu County, Kenya. MTRH is a peri-urban tertiary facility with a dedicated outpatient cardiology clinic serving an average of 320 patients per month. In 2019, the GDP per capita of Uasin Gishu County was $1 623 [14]. Most of the patients receiving care at the MTRH cardiology clinic have HF caused by valvular heart disease [11]. Written informed consent was given by all study participants. Ethical review boards at Duke University and Moi University approved the study. An independent data, safety, and monitoring board (DSMB) was also established to provide oversight.

### Participants

The study enrolled one hundred participants over a period of two years from July 2016 to June 2018. Study sample size was based on a reasonable estimate of expected recruitment rates over the study duration, as well as available capacity at the facility. Prospective study participants were identified by screening patient records. All patients over the age of 18 years, with NYHA class II and III HF symptoms were eligible. Participants with recent acute illness, inability to exercise, known arrhythmia, angina, congenital heart disease or obstructive structural heart disease were excluded. Screening for cardiac ischemia was conducted using the Master’s two step test [15] prior to study arm allocation.

### Allocation

Allocation was non-randomized. Entry into either the IBCR model or the HBCR model was on a rolling basis and based on participant preference. The IBCR arm was open to 25 participants while, in consideration of the larger geographical reach of HBCR, the HBCR arm was open to 75 participants. If either arm filled up first, subsequent participants would be allocated to the remaining slots in the open arm.

In the course of conducting the study, a higher incidence of clinically significant events, including two deaths, were observed in the HBCR arm as highlighted in the results below. Though the events were deemed not directly related to the CR intervention by the DSMB, 16 weeks into the study, further enrollment into the HBCR arm was stopped after enrollment of 31 participants. The remaining 44 participants were enrolled into an observational arm (OA).

### Rehabilitation models

IBCR sessions comprised 36 individually tailored rehabilitation sessions on a treadmill or cycle ergometer. Speed settings were adjusted to maintain a moderate level of perceived exertion using the Borg scale [16]. Target heart rates were computed at each session using Karvonen’s formula [17]. During the first four weeks, target heart rate was set to 50–60% of max heart rate (HR). During week 5–8 target heart rate was increased to 60–70% of their max HR and during week 8–12, 70–80% of their max HR. Duration of aerobic exercise was increased by 5–10 minutes at each session, with a goal of attaining 60 minutes of aerobic exercise by the end of 36 sessions.

HBCR comprised 12 individualized weekly step targets. A participant’s initial weekly step target was computed as, the average step rate attained while walking at aerobic threshold on a treadmill, multiplied by 20 minutes for seven days. HBCR participants were instructed to set aside a convenient time each day when they would exercise by walking briskly. Participants were taught to assess level of exertion based on interval measurements of heart rate and perception of moderate exertion using the Borg scale [16]. Attainment of step targets was monitored using tri-axial pedometers worn on participant’s hips. The study coordinator contacted HBCR participants weekly via phone to enquire what step count was attained and convey the new step count target. The new step target incorporated a 10% increase over and above the preceding week’s step target. The target duration of exercise was increased by 20 minutes every four weeks up to a goal of 60 minutes at the end of 12 weeks.

Every month both IBCR and HBCR participants came to the rehabilitation center for a follow-up visit where functional capacity was reassessed. During these visits, exercise coaching and behavioral modification was re-emphasized.

### Outcomes

The primary endpoint was attainment of a mean protocol adherence of at least 25%. An intention to treat approach was used for estimating any differences in adherence across arms. This proportion was based on protocol adherence of 20–50% previously reported in other studies [6,9,18].

IBCR adherence was calculated as a proportion of the 36 scheduled sessions completed by participants. HBCR adherence was calculated as a proportion of the 12 prescribed weekly step targets attained by participants. Cardiac rehabilitation was deemed feasible if participants adhered to at least 25% of scheduled activities in either model.

Secondary outcome measures were evaluated at the start of the study protocol and at monthly follow-up visits. Self-reported functional capacity was measured using the NYHA. Assessments of 6-minute walk time distance (6MWTD) was used to evaluate clinical change. Clinically significant adverse events including falls, hospital admissions and deaths were monitored.

### Statistical methods

Study data were collected and managed using REDCap electronic data capture tools hosted at Duke University [19]. Analyses were conducted using STATA software V.14. Data were expressed as number (percent), means (standard deviation [SD]) or median (interquartile range [IQR]). We used the paired T-test and ANOVA for comparisons of the means of continuous variables, the non-parametric Wilcoxon signed-rank test for comparing medians of continuous variables, and Chi-square and Fisher’s test for comparison of categorical variables. Given that the primary objective of the study was feasibility and not efficacy, any statistical testing of comparisons of means or proportions across study arms were considered exploratory. We report crude differences in mean 6MWTD and not means adjusted for possible covariate imbalance across arms because, *a priori*, we did not consider possible confounders of the treatment effect and adjustment for covariates observed to be imbalanced (i.e., age) would have likely led to residual confounding. However, we do report 6MTWD means adjusted for age, gender, and BMI, and cause of HF as a sensitivity analysis.

## Findings

**Figure 1:**
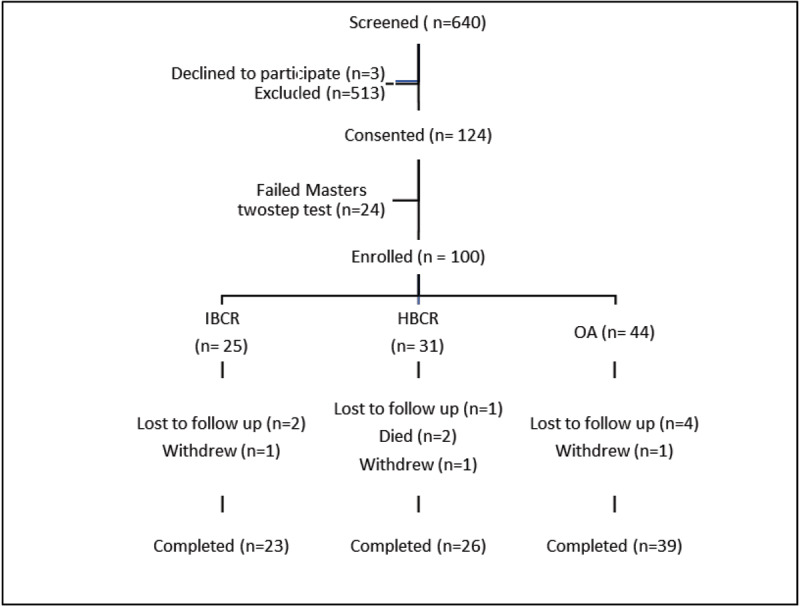
Outline of study participant allocation.

Between January and December 2017, six hundred and forty patients were screened, of whom 124 were recruited (***Figure 1***). The most common exclusion reasons were: non- limiting (NYHA class I) symptoms (n = 208), arrhythmias or pacemaker use (n = 122), structural heart disease including valvular or congenital heart disease (n = 90), geographic limitations/inflexible patient schedules (n = 22), acute decompensation or NYHA class IV symptoms (n = 20), or other comorbidities limiting ability to walk (n = 54). Twenty-four additional participants were excluded after failing a master’s two -step test due to positive ECG changes, angina, or functional limitation.

The mean age of study participants was 51 years, and 72% were female. Twenty-five participants opted to enroll into the IBCR arm, while thirty-one participants opted for the HBCR arm. Forty-four participants were enrolled into the observational arm (OA) as summarized in ***Table 1***. The study DSMB adjudicated three adverse events observed in the HBCR arm. One participant died from sepsis, one participant died as a result of HF progression and a third participant was withdrawn from the study after a stroke.

**Table 1:** Summary of participant characteristics by enrollment arm.


VARIABLE	IBCR (N = 25)		HBCR (N = 31)		OA* (N = 44)		P

Sex, male n (%)	8	32	3	10	17	39	**0.02**

Age in years: mean, (SD)	56	17	44	16	54	16	**0.01**

Weight in Kgs: pre-rehab mean, (SD)	73	22	71	17	64	14	0.07

Height in meters: mean, (SD)	162	7	163	7	164	9	0.55

BMI mean, (SD)	28	8	27	7	24	6	0.04

Waist circumference in cm: mean, (SD)	96	24	91	16	89	14	0.25

Hip circumference in cm: mean, (SD)	106	17	106	13	97	13	**0.01**

Resting heart rate bpm: mean, (SD)	71	9	71	15	77	13	0.08

Resting respiratory rate (SD)	18	3	18	3	18	3	0.58

Systolic BP in mmHg: mean, (SD)	138	17	132	21	132	20	0.42

Diastolic BP in mmHg: mean, (SD)	84	12	79	11	82	12	0.19

Ejection fraction% (SD)	50	15	49	14	45	15	0.29

**HF PHENOTYPES**	**N**	**%**	**N**	**%**	**N**	**%**	**P**

EF >50%	16	64	20	65	21	48	

EF 40-50%	4	16	3	10	7	16	0.51

EF <40%	5	20	8	26	16	36	

Rheumatic heart disease	4	16	7	23	8	18	**0.02**

Hypertensive heart disease	16	64	13	42	11	25	

Others (Ischemic, peripartum, tuberculous & unknown)	5	20	11	35	25	56	

**Overall Protocol Adherence%**	**46**	**18**	**29**	**11**	.	.	.


IBCR – institution based cardiac rehabilitation, HBCR – home based cardiac rehabilitation, OA – Observational arm. *- no evaluation of adherence since there was no intervention in the OA arm.

**Figure 2:**
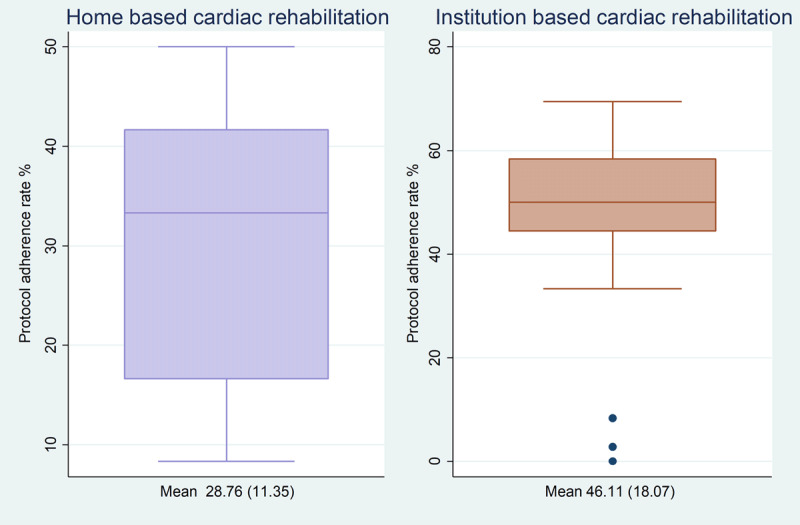
Participant Adherence according to Cardiac rehabilitation model.

Rehabilitation protocol adherence exceeded the pre-specified threshold of 25% in both the IBCR and the HBCR arms, with a mean protocol adherence rate of 46 ± 18% (22 of 25 participants) and 29 ± 11% (23 of 31 participants) respectively (***Figure 2***). Seventeen (71%) participants in the IBCR arm and eleven (41%) participants in the HBCR arm reported an improvement in one or more NYHA functional class from baseline, while the rest reported no change. Twenty (54%) participants in the OA arm noted improvement in functional class, 14 (38%) reported no change while three participants (8%) reported worsening in their NYHA class symptoms (***Table 2***).

**Table 2:** Summary of changes in functional capacity by study arm among participants who completed follow up*.


CHANGE IN NYHA CLASS	IBCR		HBCR		OA		P
			
	FREQ.	PERCENT	FREQ.	PERCENT	FREQ.	PERCENT	FISHER’S

Got worse (+1)	0	0	0	0	3	8	0.06

No change (0)	7	29	16	59	14	38	

Better (–1)	15	63	10	37	20	54	

Much better (–2)	2	8	1	4	0	0	

**CHANGE IN 6MWTD**	**MEAN(M)**	**SD**	**MEAN(M)**	**SD**	**MEAN(M)**	**SD**.	**ANOVA**

6MWTD in meters – initial	278	78	292	59	259	68	0.13

6 MWTD in meters – month 1	314	57	315	57	303	58	0.64

6 MWTD in meters – month 2	323	66	331	56	304	59	0.23

6 MWTD in meters – month 3	316	74	339	52	304	80	0.16

Change in 6 MWT distance in meters (over 3 months)	31.25	64.98	40.15	54.68	38.24	71.42	0.88

P (Paired t-test)	0.027		<0.001		0.0025		

Adjusted Change in 6MWTD *	34	27.19	45	26.06	33	21.94	0.134


6MWTD – six-minute walk time distance; NYHA- New York Heart Association, IBCR – institution based cardiac rehabilitation, HBCR – home based cardiac rehabilitation, OA – Observational arm. * The study design is underpowered to make comparative effectiveness assessments; sensitivity analysis was conducted adjusting for baseline imbalances in age, gender, BMI, and cause of heart failure.

Participant functional capacity as measured by mean change in 6-minute walk time distance (6MWTD) per study participant, increased by 31 ± 65 meters in the IBCR arm, 40 ± 55 m in the HBCR arm and 38 ± 71 meters in the observational arm (p < 0.05). There were only slight differences in the mean 6MWTD between the three arms at month one, two or three of follow up (***Table 2***). Adjustment of these mean changes in 6MWTD for age, sex, BMI, and HF cause did change somewhat, especially for the OA arm (model-adjusted marginal means: IBCR = 34.04; HBCR = 44.83; OA = 33.01). Notably, however, there was very large variation in these 6MWTD changes across participants, regardless of study arm, as indicated by the large standard deviation values (***Table 2***).

## Discussion

This study found that cardiac rehabilitation using both institution and home-based rehabilitation models, is a feasible intervention for patients with HF in Western Kenya. While feasibility is a broad construct, the feasibility of cardiac rehabilitation was considered using the “Can it work; Does it work; and Will it work?” feasibility framework, as described by Bowen et al [20]. The study focused on assessing whether rehabilitation models were feasible and utilized protocol adherence to inform practicability of the intervention. By attainment of an adherence rate of greater than 25% in both study arms, participants demonstrated that cardiac rehabilitation could be done, relative to a global adherence estimate of 20–50% [6,9,18].

Appreciably there was a difference in the proportion of participant adherence between the two models. Participants in the IBCR arm were older and attained a mean adherence rate of 46% while HBCR arm had an adherence rate of 29%, including those who withdrew or were withdrawn from the study. This difference is likely reflective of study arm self-selection by participants as well as the challenges in HBCR including imperfect monitoring devices and reporting systems as previously noted in review studies on cardiac rehabilitation adherence [7,21,22]. In this study, we further observed wide variations in adherence using patient self-reported exercise compared to device monitoring. Adherence in the HBCR arm was likely under-reported, given that participants continued to exercise even when their pedometers malfunctioned. On the other hand, higher adherence rates reported in the IBCR arm could be attributable to the influence of group social interaction. Participants in the IBCR arm were observed to check in and encourage one another, a characteristic of group social interactions noted in other cardiac rehabilitation cohorts, that may have led to improved participant adherence [6,9].

Secondarily, this study sought to shed light on whether cardiac rehabilitation “does work” in the local environment by measuring changes in NYHA class and 6MWTD as surrogate measures of intervention efficacy. Although the study was not designed to compare efficacy between the CR models, 6MWTD improved significantly across all three arms. Change in 6MWTD increased from month to month among HBCR participants, while it plateaued and decreased after the first month among IBCR participants. There was little difference in magnitude of improvement observed between study arms overall and further studies evaluating response to CR in this region are recommended (Supplementary Table 3 & 4). In studies evaluating efficacy of CR such as the “Heart Failure: A Controlled Trial Investigating Outcomes of Exercise Training (HF-ACTION)” study, patients participating in CR had a greater improvement in median 6MWTD than participants receiving usual care (20 ± 37 m vs 5 ± 32 m) [23,24]. Notably, it is still possible that CR strategies are not effective in this population or benefit only specific HF sub-populations. Compared to previously published studies of CR for HF, patients with HF in Western Kenya differ in baseline characteristics such as age, etiology of HF and, unique environmental and socio-economic constraints [23,25,26].

Ultimately, whether cardiac rehabilitation “will work” in this environment will best be assessed by long term evaluation of outcomes. Neither IBCR nor HBCR fully overcomes access and utilization barriers. Successful implementation will rely heavily on integration of existing and novel strategies that enable adaptation to clinical practice in Western Kenya. For example, this study leveraged mobile phone-based connectivity and remote monitoring to overcome some geographical access barriers in the HBCR study arm, and provided a lower cost option to CR participants. In addition, overcoming access barriers through increasing CR health literacy will likely go a long way in easing the referral and enrollment process, and further support CR adherence. In addition, development of policy and re-imbursement models that adapt to the dynamic effects of urbanization will be necessary for CR to work overtime.

### Limitations

Being a non-randomized study, confounding caused by self-selection into treatment arm likely biased any comparison of adherence to cardiac rehabilitation by treatment arm. And although estimates adjusted for age and sex did not appreciably change study results, this does not preclude the possibility of residual confounding by unmeasured factors. Whereas the study assessed feasibility based on ability of participants to adhere to CR protocols, adherence in this study was likely influenced by participants ability to choose which rehabilitation model to take part in. In addition, the study design and small sample size does not allow for meaningful comparisons in effect size across study arms, or certainty that CR will yield substantive improvements in functional capacity.

## Conclusion

In conclusion, cardiac rehabilitation is a feasible intervention for patients with HF in Western Kenya. Whereas some CR participants in both rehabilitation models demonstrated improvement in functional capacity, the effectiveness of cardiac rehabilitation in this population remains unknown. In addition to development of innovative ways to support of CR adherence, further studies are needed to evaluate whether cardiac rehabilitation is effective in this population as well as determination of long-term efficacy and safety profiles.

## Data Accessibility Statement

The datasets used and/or analyzed during the current study are available from the corresponding author on reasonable request.

## Additional Files

The additional files for this article can be found as follows:

10.5334/aogh.3392.s1 Supplementary Table 3. Supplementary table of baseline participant characteristics by protocol adherence.

10.5334/aogh.3392.s2 Supplementary Table 4. Supplementary table showing change in 6MWT distance at month 1, 2 and 3 of follow up.

## Figures and Tables

**Table:** 

6MWTD	6-minute Walk Time Distance
CR	Cardiac Rehabilitation
DSMB	Data Safety and Monitoring Board
HBCR	Home Based Cardiac Rehabilitation
HR	Heart Rate
IBCR	Institution Based Cardiac Rehabilitation
IQR	Interquartile Range
MTRH	Moi Teaching and Referral Hospital
NYHA	New York heart association
OA	Observational arm
SD	Standard Deviation
