# Supplementary material for: Feasibility of Cardiac Rehabilitation Models in Kenya
Source: Ann Glob Health. 2022 Jan 18;88(1):7. doi: 10.5334/aogh.3392 (PMC8782083; doi:10.5334/aogh.3392)
Supplement: Supplementary Table 3. — Supplementary table of baseline participant characteristics by protocol adherence. [file agh-88-1-3392-s1.pdf]

Table 3 **Summary of participant characteristics by enrollment arm subcategorized by participant adherence rates.**

	IBCR					HBCR				
**Variable**	**Adherent (>25%) n = 22**		**Non adherent n =3**		**Fisher/ Wilcoxon test**	**Adherent (>25%) n =23**		**Non adherent n= 8**		**Fisher/ Wilcoxon test**
	**Mean**	**SD**	**Mean**	**SD**	**P**	**Mean**	**SD**	**Mean**	**SD**	**P**
Sex, male n (%)	6	27	2	67	0.23	2	9	1	13	1.00
Age in years: mean, (SD)	56	18	56	18	0.92	40	15	55	11	0.01
Weight in Kgs: pre-rehab mean, (SD)	72	22	82	22	0.50	68	17	80	16	0.08
Height in meters: mean, (SD)	162	7	164	4	0.84	164	6	159	7	0.09
BMI mean, (SD)	27	8	31	10	0.66	25	7	32	6	0.01
Waist circumference in cm: mean, (SD)	95	24	106	24	0.72	87	17	103	6	<0.01
Hip circumference in cm: mean, (SD)	105	14	117	30	0.65	103	13	116	11	0.01
Resting heart rate bpm: mean, (SD)	71	10	73	6	0.80	71	14	71	17	0.97
Resting respiratory rate (SD)	18	3	16	2	0.06	18	4	17	2	0.36
Systolic BP in mmHg: mean, (SD)	139	15	132	32	0.71	136	21	122	17	0.08
Diastolic BP in mmHg: mean, (SD)	85	12	82	14	0.80	81	11	72	11	0.05
Ejection fraction % (SD)	51	14	38	32	0.72	51	13	41	16	0.17
**HF phenotypes**	**n**	**%**	**n**	**%**	**P**	**n**	**%**	**n**	**%**	**P**
EF >50%	14	64	2	67		17	74	3	38	
EF 40-50%	4	18	.	.	1.00	3	13	.	.	0.03
EF <40%	4	18	1	33		3	13	5	63	
Rheumatic heart disease	3	14	1	33		7	30	.	.	
Hypertensive heart disease	14	64	2	67	0.50	7	30	6	75	0.06
Others (Ischemic, peripartum; tuberculous & unknown)	5	23	.	.		9	39	2	25	
**6-MWTD Change in meters (SD)**	31	66	40	71	0.80	40	59	41	34	0.69

6MWTD – six-minute walk time distance; NYHA- New York Heart Association, HF- Heart failure, EF- Ejection Fraction; IBCR - institution based cardiac rehabilitation, HBCR - home based cardiac rehabilitation, OA - Observational arm
