# Supplementary material for: Feasibility of Cardiac Rehabilitation Models in Kenya
Source: Ann Glob Health. 2022 Jan 18;88(1):7. doi: 10.5334/aogh.3392 (PMC8782083; doi:10.5334/aogh.3392)
Supplement: Supplementary Table 4. — Supplementary table showing change in 6MWT distance at month 1, 2 and 3 of follow up. [file agh-88-1-3392-s2.pdf]

Table 4: **Comparison of 6 Minute walk time distance by study arm and protocol adherence using change analysis**

**A. IBCR**	Combined (adherent & non adherent)		Adherent (>25%) n=22		Non-adherent n=3	
**Change in 6MWTD Over Time**	**Mean(m)**	**SD**	**Mean(m)**	**SD**	**Mean(m)**	**SD**
6MWTD in meters – initial	278	78	282	71	248	141
6 MWTD in meters – month 1	314	57	314	57	.	.
6 MWTD in meters – month 2	323	66	323	66	.	.
6 MWTD in meters – month 3	316	74	313	64	350	198
Change in 6 MWT distance in meters (over 3 months)	31	65	31	66	40	71

**B. HBCR**	Combined (adherent & non adherent)		Adherent (>25%) n= 23		Non-adherent n=8	
**Change in 6MWTD**	**Mean(m)**	**SD**	**Mean(m)**	**SD**	**Mean(m)**	**SD**
6MWTD in meters – initial	304	55	301	52	258	60
6 MWTD in meters – month 1	324	44	321	47	283	86
6 MWTD in meters – month 2	341	47	331	46	288	78
6 MWTD in meters – month 3	342	54	339	52	327	42
Change in 6 MWT distance in meters (over 3 months)	40	59	40	55	41	34
